# The enzymatic epimerization of deoxynivalenol by *Devosia mutans* proceeds through the formation of *3-keto-*DON intermediate

**DOI:** 10.1038/s41598-017-07319-0

**Published:** 2017-07-31

**Authors:** Yousef I. Hassan, Jian Wei He, Norma Perilla, KaiJie Tang, Petr Karlovsky, Ting Zhou

**Affiliations:** 1 Guelph Research and Development Centre, Agriculture and Agri-Food Canada, Guelph, Ontario Canada; 2 School of Environmental Sciences, University of Guelph, Guelph, Ontario Canada; 3 Micotox Ltd, Bogota, Colombia; 4 Molecular Phytopathology and Mycotoxin Research, Georg-August-University Göttingen, Göttingen, Germany

## Abstract

The enzymatic detoxification of deoxynivalenol (DON) is a promising mitigation strategy for addressing this mycotoxin contamination of cereal grains. A recently described bacterium, *Devosia mutans* 17-2-E-8, capable of transforming DON into its non-toxic stereoisomer *3-epi*-DON, holds promise for the development of such applications. Earlier observations suggested that DON epimerization proceeds via a two-step catalysis with *3-keto-*DON as an intermediate. The results of this study indicate that NADPH is required for DON epimerization by cell-free protein extracts of *D*. *mutans*, while high concentrations of glucose and sucrose have a suppressive effect. Chemically synthesized *3-keto-*DON incubated with *D*. *mutans* protein fractions enriched by ammonium sulfate precipitation at 35–55% saturation selectively reduced *3-keto-*DON to *3-epi*-DON, but fell short of supporting the complete epimerization of DON. In addition, seven *Devosia* species investigated for DON epimerization were all able to reduce *3-keto-*DON to *3-epi*-DON, but only a few were capable of epimerizing DON. The above observations collectively confirm that the enzymes responsible for the oxidation of DON to *3-keto-*DON are physically separate from those involved in *3-keto*-DON reduction to *3-epi-*DON. The enzymatic nature of DON epimerization suggests that the process could be used to develop genetically engineered crops or microorganisms, ultimately reducing foodborne exposure of consumers and farm animals to DON.

## Introduction

Food and feed commodities contaminated with deoxynivalenol (DON) pose a health risk to both humans and animals^1–4^ and form an international trade-barrier for cereal grain products. Devastating agricultural and economical losses, estimated at $5 billion within North America alone, are associated with *Fusarium* infection and DON contamination in corn and wheat products. Enzymatic detoxification is a promising strategy to address the contamination of cereal grains with DON^5^. Previous studies reported the epimerization of DON to *3-epi*-DON in Gram-negative and Gram-positive bacterial isolates such as *Devosia mutans* 17-2-E-8 and *Nocardioides* sp. strain WSN05-2^6–9^, as well as the oxidation of DON to *3-keto-*DON by a Gram-negative bacterial strain^10^ and an uncharacterized bacterial culture^11^. The recent large-scale purification of *3-epi*-DON^7^ allowed for studies of its toxicity, which showed that *3-epi*-DON was essentially non-toxic^12^. This finding, combined with the ability of DON epimerization^6, 13, 14^ to proceed under aerobic conditions, offers promise for developing new management strategies to control this mycotoxin.

**Figure 1:**
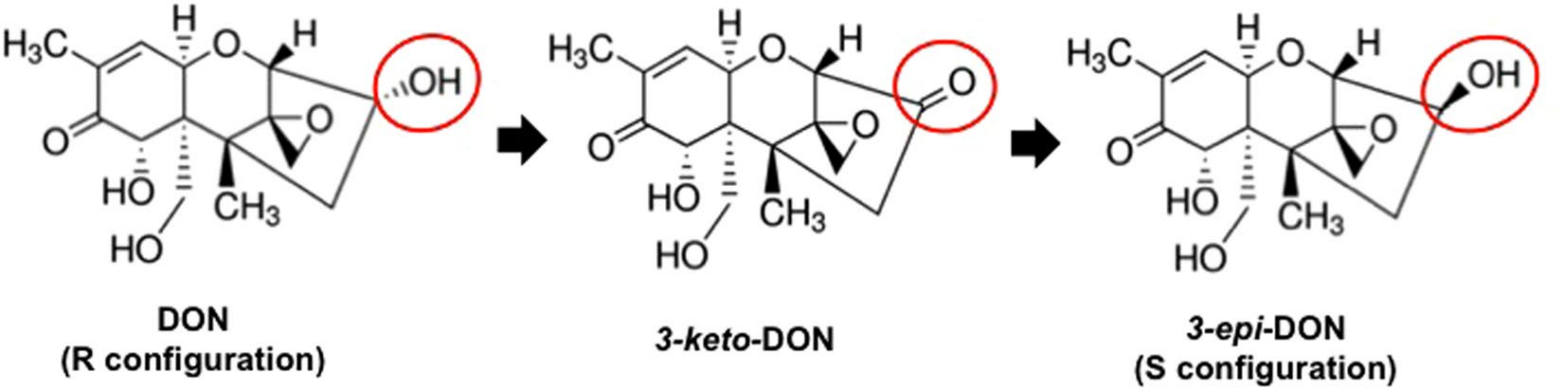
The two-step bacterial transformations of deoxynivalenol (DON) to *3-epi*-DON proceeds through the formation of *3-keto-*DON intermediate. DON biotransformation to *3-epi*-DON in different Gram positive and Gram negative bacterial isolates (including *Devosia mutans*) is predicted to proceed through two separate enzymatic steps. The first oxidation step leads to the formation of *3-keto-*DON intermediate while the second reduction step leads to *3-epi-*DON accumulation.

The mechanism by which bacterial epimerization of DON occurs is still unknown. Previous observations indicate it involves a two-step process, with *3-keto-*DON as an intermediate (Fig. 1), though this has not yet been conclusively demonstrated^5, 7^. In this work, we chemically synthesized *3-keto-*DON and monitored its transformation by cultures of seven *Devosia* species and by cell-free lysates of *D*. *mutans* 17-2-E-8. The results clearly show that the epimerization of DON does in fact proceed in two steps that can be physically separated.

## Results

### Synthesis of *3-keto-*deoxynivalenol

**Figure 2:**
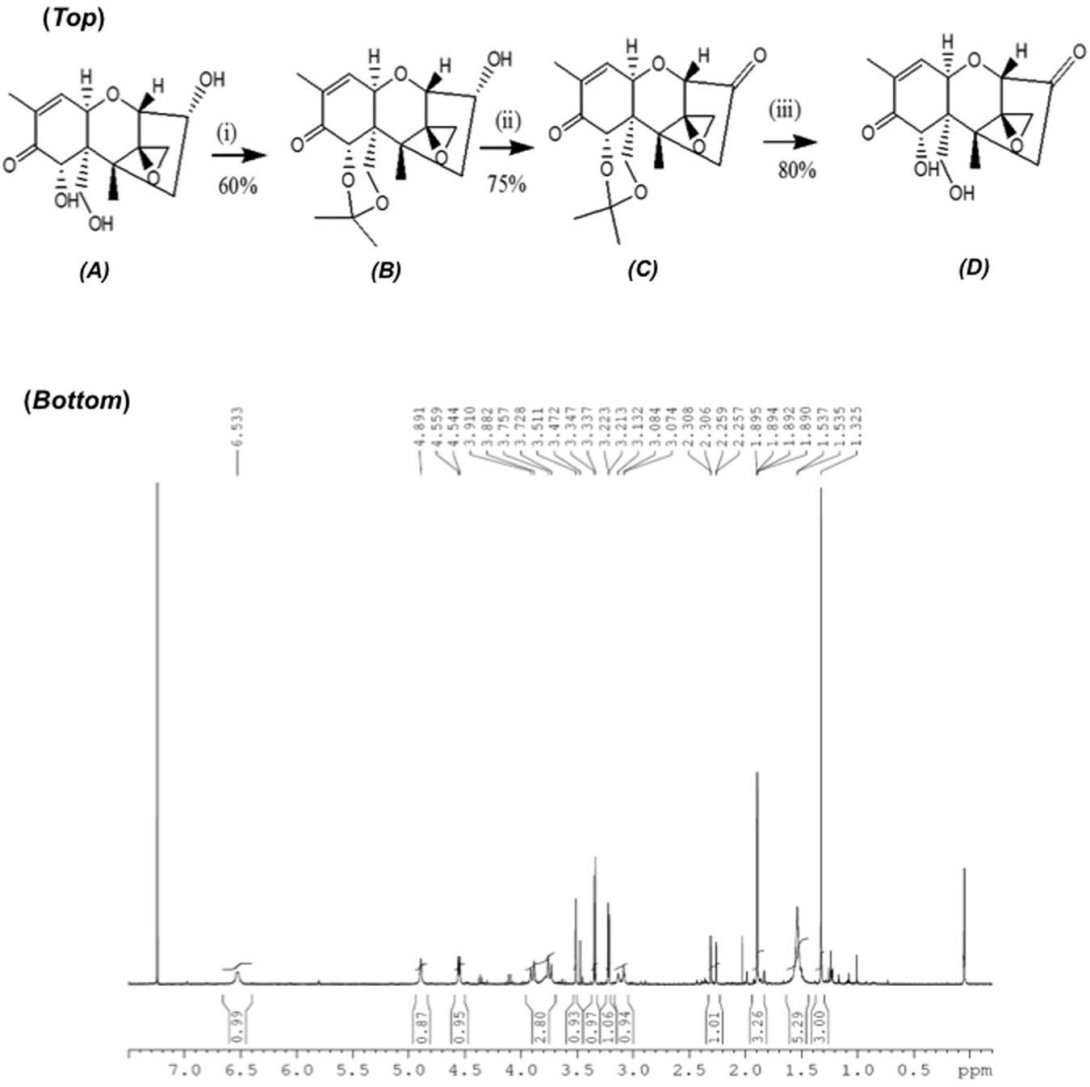
The chemical synthesis of *3-keto-*DON and the retrieved proton (^1^H) NMR spectra of the synthesized compound. (*Top*): Chemical synthesis of *3-keto-*DON (**D**) encompasses protecting the 7,15-dihydroxyl groups of DON (**A**) by using 2,2-dimethoxypropane (i) before oxidizing the secondary hydroxyl (ii) in a later phase. The protecting-groups were removed later through a hydrolysis initiated with TFA (iii) to finally obtain the desired *3-keto-*DON intermediate (**D**). (*Bottom*): The obtained chemical shifts of ^1^H-NMR signals of the chemically synthesized *3-keto-*DON are identical with previously published data for the same compound^10, 15^. A detailed discussion of the spectra is found within the results section. An overall yield close to 36% was achieved combined with purity levels close to 95%.

The 7,15-dihydroxyl groups of DON (Fig. 2-*top*-*A*) were protected with 2,2-dithoxypropane (i) while the secondary hydroxyl (Fig. 2-*top*-*B*) was oxidized with TPAP-NMMO (ii) to form the *3-*ketone derivative (Fig. 2-*top*-*C*) as described in Materials and Methods. After removal of the protecting-groups (iii), the ^1^H-NMR analysis confirmed the identity and purity of the obtained *3-keto-*DON (Fig. 2-*top*-D). The obtained NMR spectrum for the purified compound (shown in Fig. 2-*bottom*) was: ^1^HNMR: (Bruker DPX-400 MHz, CDCl3) δ1.31 (3H, s, H-14), 1.89 (3H, br s, H-16), 2.28 (br d, *J*_AB_ = 19 HZ, H-4A) 3.07 (d, *J*_AB_ = 4 HZ, H-13A), 3.21 (d, *J*_AB_ = 19 HZ, H-4B), 3.33 (d, *J*_AB_ = 4, H-13B), 3.51 (s, H-2), 3.76 (d, *J*_AB_ = 12 HZ, H-15A), 3.91 (d, *J*_AB_ = 12 HZ, H-15B), 4.54 (d, *J*_11,10_ = 6, H-11), 4.89 (br s, H-7), 6.53 (br d, *J*_10,11_ = 6, H-10). The obtained chemical shifts of ^1^H-NMR signals of *3-keto-*DON (Fig. 2-*top-D*) were identical with previously published data for the same compound^10, 15^. The overall yield was 36% while the purity was >95%.

### *3-keto-*DON concurs with *3-epi-*DON and transiently accumulates during the isomerization of DON

**Figure 3:**
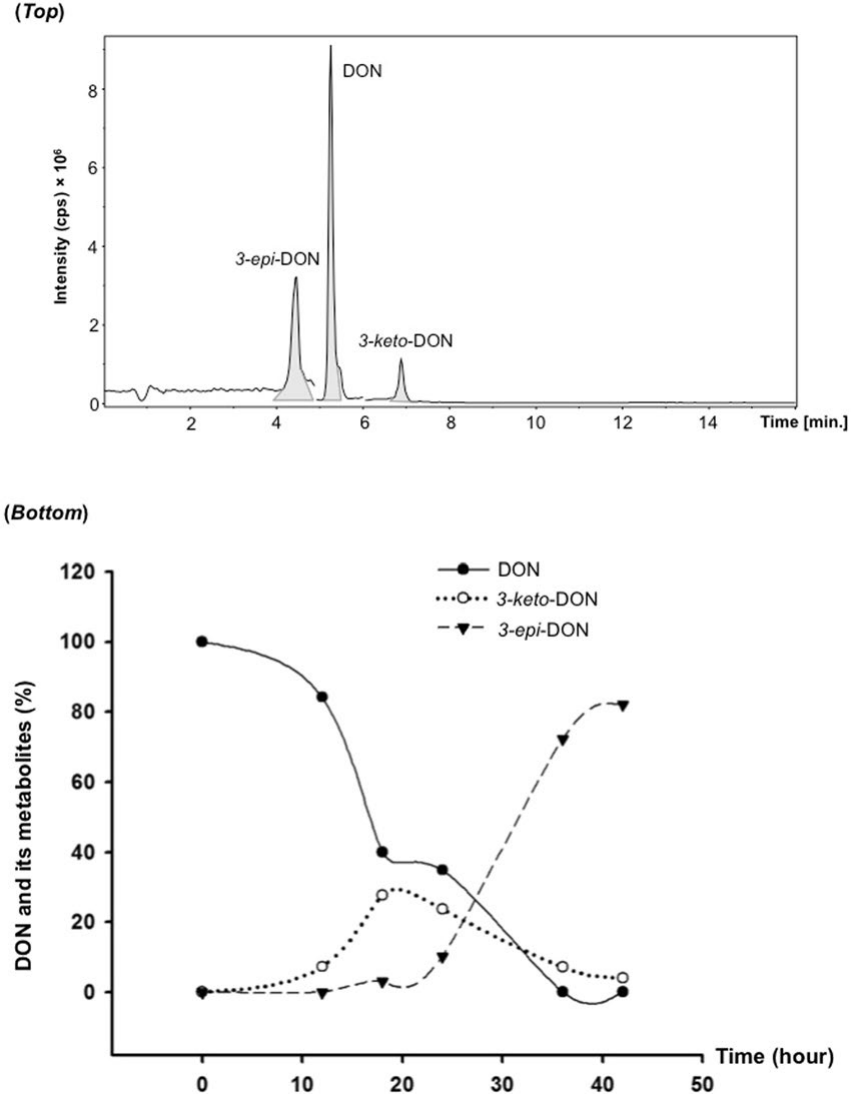
The chromatographic separation of DON, *3-keto-*DON and *3-epi-*DON by LC-MS/MS and DON epimerization dynamics in *Devosia mutans* 17-2-E-8 cultures growing in Corn Meal broth (CMB). (*Top*): The chromatographic analysis of DON, *3-keto-*DON and *3-epi-*DON using LC-MS/MS platform. The extracted ion chromatograms for transitions m/z 296.5 > 249.1 for DON as well as *3-epi*-DON and m/z 294.9 > 247.1 for *3-keto-*DON are shown. (*Bottom*): Both the disappearance of DON and the appearance/accumulation of *3-keto*-DON and *3-epi*-DON were monitored in *D*. *mutans* cultures up to 42 h as reported within the Materials and Methods section.

The simultaneous chromatographic separation of DON, *3-keto-*DON, and *3-epi*-DON (Fig. 3-*top*) confirmed the feasibility and validity of the above chemical synthesis as a basis for our bacterial/enzymatic biotransformation studies.

Actively growing cells of *D*. *mutans* 17-2-E-8 were able to transform DON to *3-epi*-DON as shown in Fig. 3-*bottom*. *3-keto-*DON was detected during *3-epi*-DON formation, with highest concentrations detected between 18–25 h after inoculation. After 40 hours, both DON and *3-keto-*DON concentrations fell below 5–7% of the original concentration of DON while *3-epi*-DON concentration reached 80–85% of the original concentrations of DON (Fig. 3-*bottom*).

**Figure 4:**
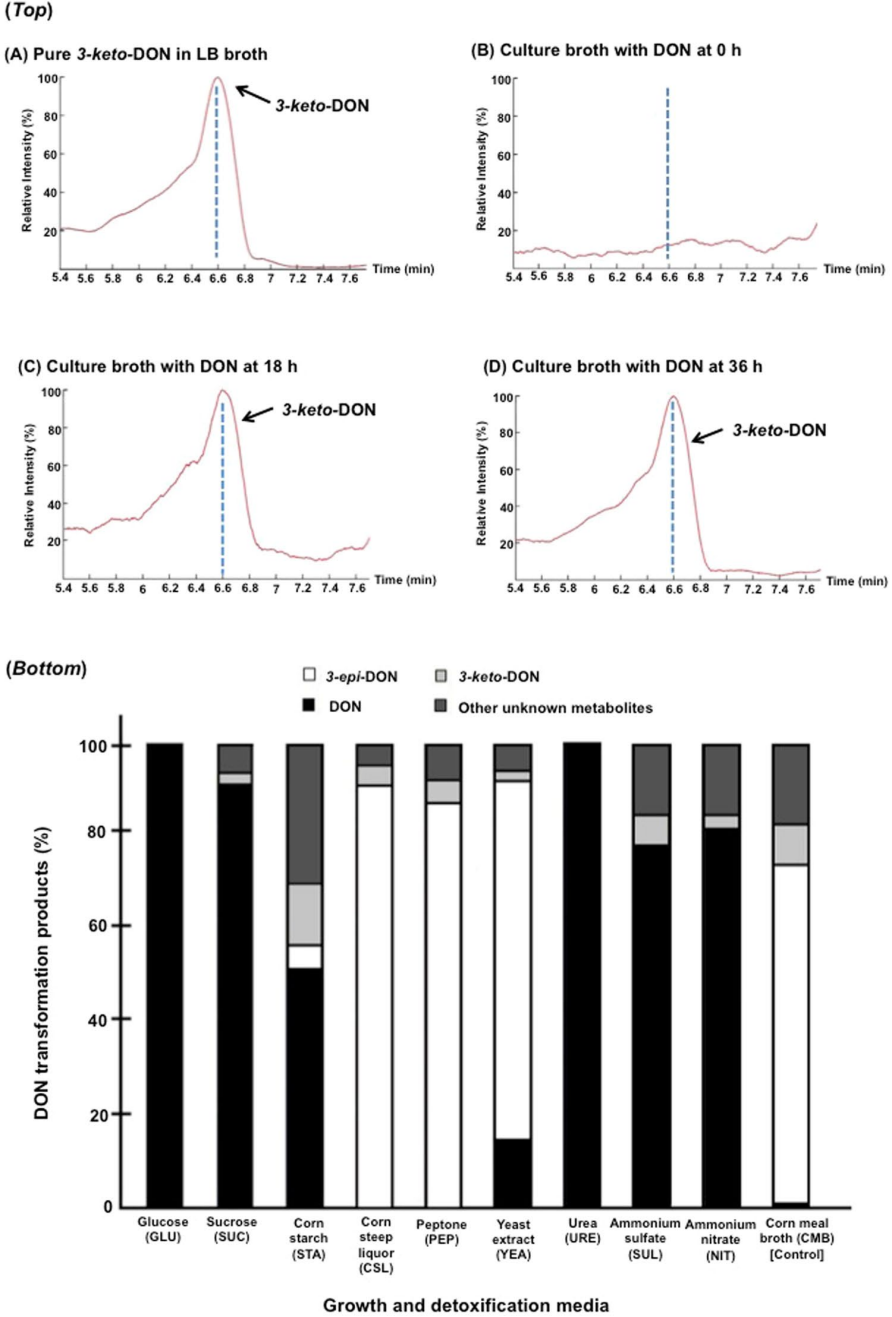
The detection of *3-keto-*DON in *Devosia mutans* 17-2-E-8 bacterial cultures and *3-keto-*DON and *3-epi-*DON concurrence during *Devosia mutans* 17-2-E-8 assisted-epimerisation of DON. (*Top*): The chromatographic separation of *3-keto-*DON obtained from depleted cultures of *Devosia mutans* 17-2-E-8 at 0 h, 18 h, and 36 h (*panel B*, *C*, *and D*) in comparison to *3-keto-*DON standard that was chemically synthesized (*panel A*). The analysis was conducted using LC-MS/MS by monitoring mass transition m/z 294.9 > 247.1 as described in the Materials and Methods section. (*Bottom*): Different media preparations containing: glucose, sucrose and corn starch (carbon sources); corn steep liquor, peptone, yeast extract, and urea (organic nitrogen sources); ammonium sulfate and ammonium nitrate (inorganic nitrogen sources) were tested for DON epimerization. Every broth that supported DON epimerization showed the concurrence of *3-keto-*DON at the same time. While experiments were conducted in triplicates to confirm the observed tendencies, no quantitative statistical inferences between different broths are suggested.

The identity and accumulation of *3-keto-*DON in *D*. *mutans* 17-2-E-8 cultures (Fig. 4-*top*- *C & D panels*) supplemented with DON (as substrate) was confirmed by LC-MS/MS through comparison with the chemically synthetized standard (Fig. 4-*top-A*).

In order to reveal the influence of nutrients (carbon and nitrogen sources) on the epimerization process and on *3-keto-*DON accumulation, different media formulations were evaluated. Some media formulations did not support the epimerization process, as indicated by a lack of *3-epi-*DON accumulation, however, in all those that did, including corn meal broth (CMB), corn starch (STA), corn steep liquor (CSL), peptone (PEP), and yeast extract (YEA), detectable amounts of *3-keto-*DON were observed (Fig. 4-*bottom*). Moreover, *3-keto-*DON was also detected in certain media formulations that did not support the full epimerization process, such as sucrose (SUC), ammonium sulfate (SUL), and ammonium nitrate (NIT) (Fig. 4-*bottom*), indicating a clear segregation between DON oxidation to *3-keto-*DON and *3-keto-*DON reduction to *3-epi-*DON. More importantly and while no statistical inferences are being made between different media, it appears that other DON metabolites are indeed accumulating within the studied media/cultures, in some cases accounting for up to 30% of final transformation products (as the case of corn starch for example) (Fig. 4-*bottom*).

### DON epimerization by *Devosia mutans* 17-2-E-8 is a multi-step enzymatic process enhanced by cofactor NADP(H)

**Figure 5:**
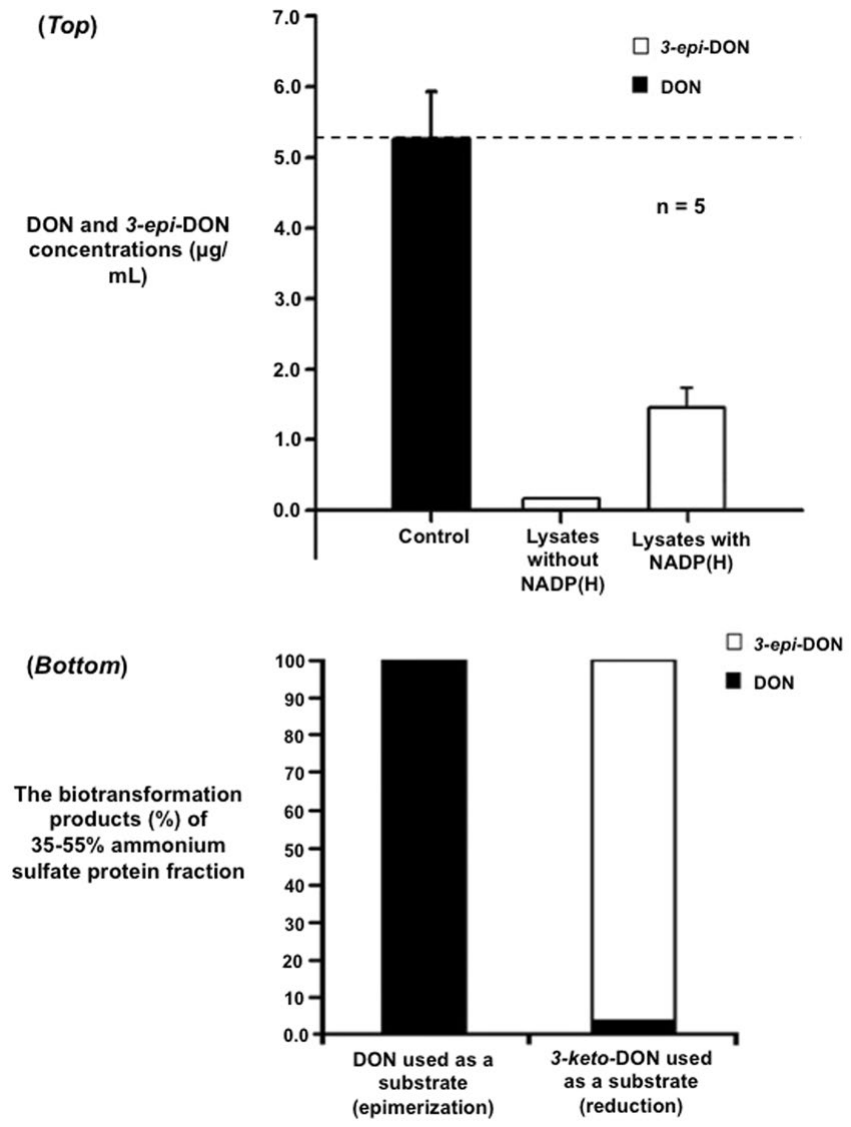
DON epimerization in cell-free lysates of *Devosia mutans* and the enzymatic activity of selected ammonium sulfate protein fractions of *Devosia mutans*. (*Top*): Whole cell-free lysates of bacteria (n = 5) were incubated for two hours with DON (as substrate) without any added cofactors or with NADP(H) at 100 µM. Concentrations of DON and *3-epi-*DON were determined by HPLC-MS/MS methods. (*Bottom*): The 35–55% ammonium sulfate protein fraction prepared as reported earlier in the Materials and Methods section was tested for the enzymatic activity by adding DON or *3-keto*-DON (as substrates) separately with a source of NADP(H). After an overnight incubation, the concentrations of DON, *3-keto-*DON and *3-epi-*DON were determined by HPLC-MS/MS methodology. While experiments were conducted in triplicates to confirm the observed tendencies, no quantitative statistical inferences between reactions are suggested for the bottom part of the figure.

Incubation of DON with cell-free lysates of *D*. *mutans* 17-2-E-8 completely eliminated DON in solution, though only a small fraction of metabolized DON was converted to *3-epi-*DON (Fig. 5-*top*). The addition of NADP(H) enhanced the accumulation of *3-epi-*DON by 15-fold, increasing its detected concentrations from 0.1 µg/mL to 1.5–1.6 µg/mL within the diluted analytical samples and showing that NADP(H) acts as a cofactor in the enzymatic production of *3-epi-*DON. The disappearance of DON from lysates not supplemented with NADP(H) (Fig. 5-*top*) without the production of equimolar amounts of *3-epi-*DON further supported the notion that the transformation of DON into *3-epi*-DON proceeds via a transient intermediate, *3-keto*-DON in this case, but also suggested the formation of some other unknown metabolites.

Protein fractionation of the *D*. *mutans* cell-free lysate by ammonium sulfate precipitation was undertaken to physically separate the enzymatic activities involved in DON transformation. Protein fractions corresponding to 35–55% ammonium sulfate saturation supported the regioselective reduction of *3-keto-*DON to *3-epi-*DON but not DON (Fig. 5-*bottom*). When DON was provided as a substrate, it remained intact (Fig. 5-*bottom*) during the incubation with the above fraction. This result corroborated our hypothesis that the epimerization of DON was a two-step process. The above protein fraction contained the enzymatic activity needed to reduce *3-keto-*DON into *3-epi-*DON while lacking the enzymes responsible for the oxidation of DON into *3-keto-*DON.

### All tested *Devosia* species reduce *3-keto-*DON to *3-epi-*DON but only few are capable of epimerizing DON

**Figure 6:**
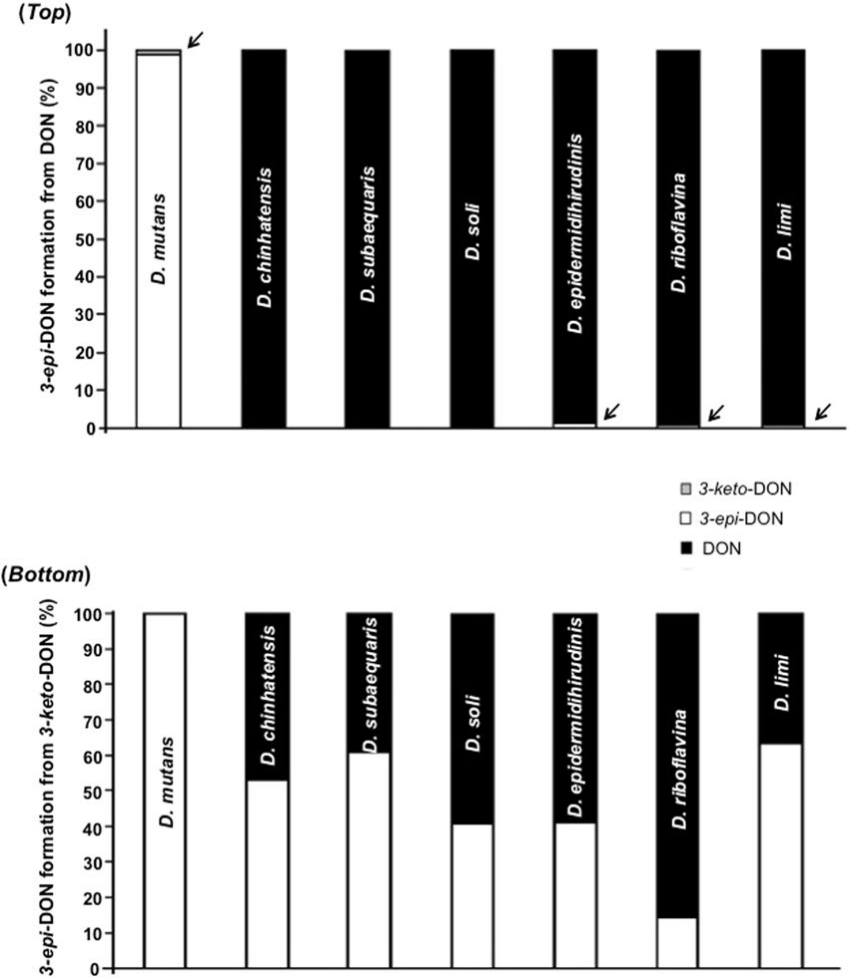
The ability of different *Devosia* type-strains to epimerize DON or to selectively reduce *3-keto-*DON to *3-epi-*DON. The simultaneous oxidation and reduction of the C3 carbon (epimerization) in DON (*Top*) or the selective reduction of *3-keto-*DON to *3-epi-*DON (*Bottom*) were tested after growing the pure isolates within the respective media preparations recommended by type-strains suppliers (DMSZ & ATCC culture collections). The concentrations of DON, *3-keto-*DON, and *3-epi*-DON were monitored as reported within the Materials and Methods section (after 24 h incubations). While experiments were conducted in triplicates to confirm the observed tendencies, no quantitative statistical inferences between strains are suggested. The incorporated arrows highlight the presence of traces of different DON metabolites.

If DON epimerization follows a two-step enzymatic process, it is hypothetically plausible that certain *Devosia* isolates may be capable of performing only the first enzymatic step, oxidizing DON to *3-keto-*DON, or only the second step, reducing *3-keto-*DON into *3-epi-*DON^10, 11^. Screening of type strains of six additional *Devosia* species confirmed this assumption (Fig. 6). Only *D*. *mutans* 17-2-E-8 supported an appreciable epimerization of DON into *3-epi-*DON (Fig. 6-*top*), while other investigated *Devosia* species either did not demonstrate the ability to epimerize DON, such as *D*. *chinhatensis*, *D*. *subaequaris* and, *D*. *soli*, or showed the accumulation of minute traces of *3-epi-*DON (Fig. 6-*top with arrows*).

On the other hand, all tested *Devosia* strains reduced *3-keto-*DON into *3-epi*-DON, though the extent of the conversion differed among these strains (Fig. 6-*bottom*). These results indicate that, unlike *D*. *mutans* 17-2-E-8, the other examined *Devosia* species possess little to no ability to oxidize DON into *3-keto-*DON, and confirmed the physical separation of DON-oxidation and DON-reduction functionalities needed for the complete epimerization. Interestingly, most of the investigated *Devosia* species were also able to reduce *3-keto-*DON back to DON (black color) as shown in Fig. 6-*bottom*.

### Molecular modeling and thermodynamic stability of *3-keto-*DON and *3-epi-*DON

Quantum mechanical calculations estimated the free energy of DON at −4158.4 kcal/mol, while the free energy of *3-epi-*DON and *3-keto-*DON were −4162.1 kcal/mol and −4033 kcal/mol, respectively. Conversion of DON to *3-epi-*DON is therefore a thermodynamically slightly favored process. According to their standard free energy, a mixture of DON and *3-epi-*DON at thermodynamic equilibrium under standard conditions would consist of 44.6% DON and 55.4% *3-epi-*DON. The observation that *D*. *mutans* 17-2-E-8 cultures converted DON into *3-epi-*DON quantitatively (Fig. 6-*top*) makes the involvement of a direct enzymatic epimerization by deprotonation/re-protonation unlikely. The transformation appears to be driven by an exothermic process that may be coupled to either the first or second step of the postulated two-step mechanism, with *3-keto-*DON serving as a transformation intermediate^5^.

**Figure 7:**
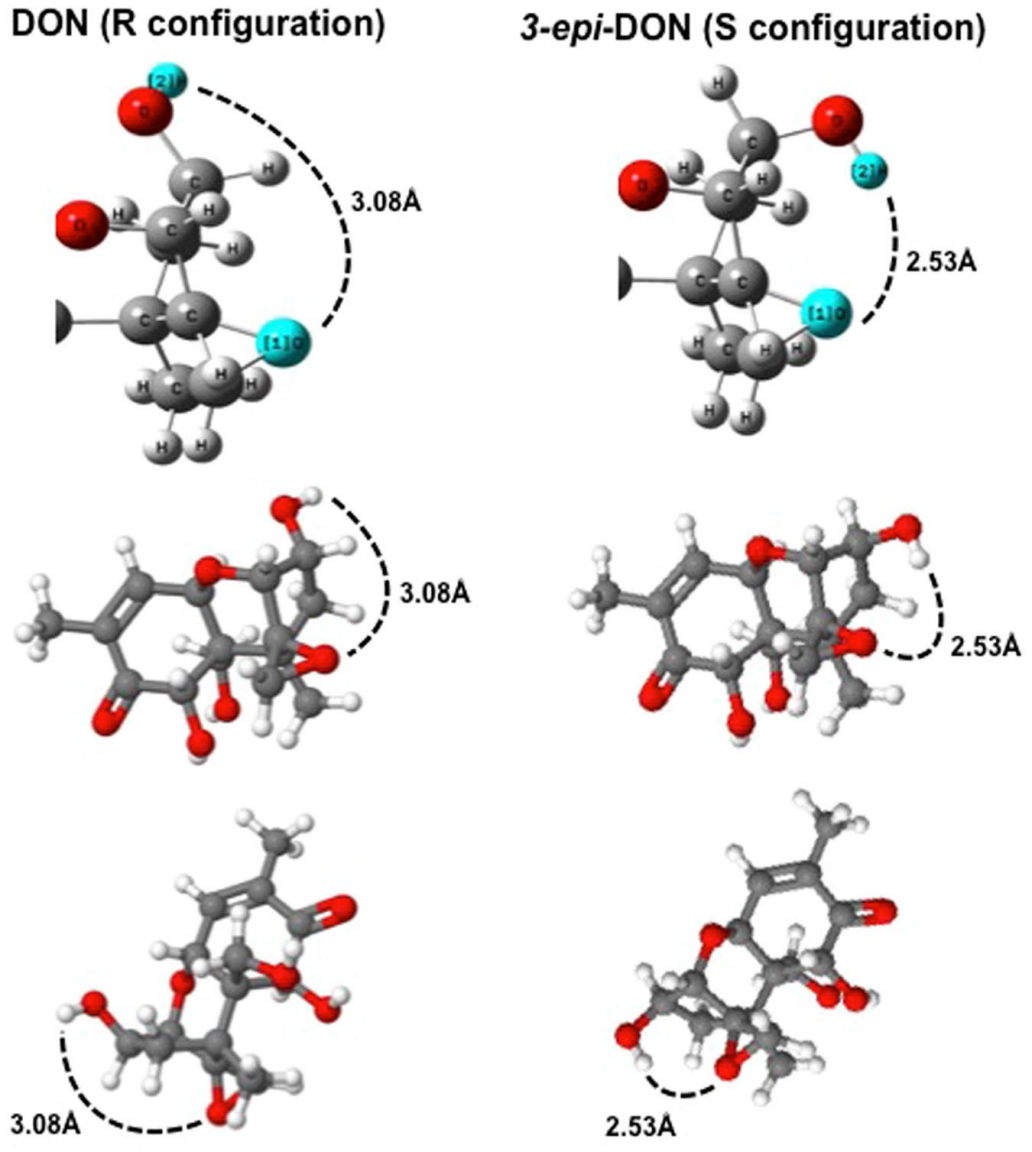
Molecular modeling of DON and *3-epi-*DON. The molecular modeling of DON (*right panel*) and *3-epi-*DON (*left panel*) chemical structures was achieved by Hyperchem 8.0. The decreased distance between the hydrogen atom of the C3 hydroxyl group and the oxygen atom of the epoxide group in *3-epi-*DON (2.5 Å) in comparison to DON (3.1 Å) suggests the possibility of an intramolecular hydrogen bond formation that contributes to *3-epi-*DON stability and influences its ribosomal binding capacity^28^.

Molecular modeling revealed that the distance between the hydrogen atom of the C3 hydroxyl group and the oxygen atom of the epoxide group decreased from 3.1 Å in DON (Fig. 7-R *configuration*) to 2.5 Å for *3-epi-*DON (Fig. 7-S *configuration*), which allows for the formation of an intramolecular hydrogen bond, potentially stabilizing the epoxide group. Apart from the loss of hydrogen bonding between the hydroxyl group of C3 and the RNA moiety inside the peptidyl transferase pocket of the ribosome, the formation of an intramolecular hydrogen bond would prevent a hydrogen bonding between the epoxide group and uracil 2873, which is essential for the inhibition of eukaryotic ribosomes by trichothecenes^16^.

## Discussion

Deoxynivalenol is a commonly detected contaminant of wheat and corn products around the globe^17^. The widespread occurrence of this mycotoxin, coupled with the adverse effects resulting from its consumption on human health and animal productivity^2–4^, has promoted intensive research efforts to identify means of inactivation^5, 13, 14, 18^. The discovery of aerobic bacteria capable of detoxifying DON through epimerization^6, 9, 19^ was followed by the purification of the transformation byproducts^7^, the assessment of their toxicity^12^, and the suggestion of a two-step mechanism of epimerization^5^. The past five years witnessed the isolation of many DON-detoxifying *Devosia* spp. on three continents^5, 8, 20, 21^, surpassing the first fifteen years of research after the genus *Devosia* was separated from *Pseudomonas* ^22^.

The development of empirical applications based on the detoxification activity of *Devosia* spp. requires a good understanding of the enzymatic reactions involved. The work presented here addresses gaps in knowledge related to DON epimerization by *D*. *mutans* 17-2-E-8.

The proposed two-step mechanism of DON epimerization by *D*. *mutans* via oxidation to *3-keto-*DON, followed by reduction to *3-epi*-DON, was confirmed in several ways. Firstly, comparison of the conversion rate achieved by intact bacteria with the theoretical ratio of DON and *3-epi-*DON in a thermodynamic equilibrium showed that the transformation is coupled to yet unknown exothermic reaction. This excluded the involvement of deprotonating epimerases with reactive sp2 intermediates. Secondly, the activity responsible for the reduction of *3-keto-*DON to *3-epi*-DON was separated from the oxidation of DON to *3-keto-*DON by ammonium sulfate fractionation of cell-free extracts. Finally, comparison of the activities of seven *Devosia* species towards DON and *3-keto-*DON showed that, while all strains were able to reduce *3-keto-*DON into *3-epi*-DON to some extent, not all were able to oxidize DON into *3-keto-*DON, as demonstrated by a lack of complete epimerization. Hence, this finding establishes the physical separation of these two enzymatic functionalities within the reported *Devosia* strains.

The epimerization of DON to *3-epi*-DON requires NADP(H), indicating the involvement of dehydrogenases/oxidoreductases in the process. NAD rather than NADP is used as a cofactor by most known bacterial catabolic dehydrogenases; NADP-dependent dehydrogenases are often involved in biosynthetic pathways. Because all seven of the *Devosia* strains tested possessed the *3-keto-*DON to *3-epi*-DON reducing activity, despite the fact that several originate from environments where DON is not expected to be present, it is likely that the natural substrate of the enzyme is not *3-keto-*DON but an intermediary metabolite produced by *Devosia* spp. that possesses a ketone group on a rigid skeleton resembling the constrained cyclopentane ring of trichothecenes^23–25^. The identification of the enzyme responsible for the reduction of *3-keto-*DON to *3-epi*-DON and the localization of its gene, perhaps within a biosynthetic pathway, might help unravel the biological function of this activity. The above observation also supports our hypothesis of a two-step enzymatic transformation of DON to *3-epi-*DON, especially in relation to the E3–39 isolate that was identified by Shima *et al*.^10^ and reportedly only capable of oxidizing DON to *3-keto-*DON. While the isolate was initially classified under the *Agrobacterium–Rhizobium* group^10^, it was found later to be most closely related to the *Devosia* genus based on its 16S rRNA gene sequence analyses^8^.

In contrast to the reduction reaction of *3-keto-*DON to *3-epi-*DON, little is known about the oxidation of DON to the *3-keto-*DON intermediate. The confinement of this activity to only few *Devosia* species is intriguing, and comparative genome analysis of these strains might be helpful in elucidating the genetic basis of the oxidation of DON to *3-keto-*DON by *D*. *mutans*. According to previously published reports, bacterial strains capable of oxidizing DON to *3-keto-*DON have been found on three continents^5, 8–11^. While some of the strains isolated in Japan do not belong to the genus *Devosia* ^9, 10^, one strain found in Germany^5, 11^ and another strain found in Japan^8^ are *Devosia* spp., and might even belong to the species *Devosia mutans*.

The high stereoselectivity of the reduction of *3-keto-*DON by *D*. *mutans* is important for its use in the detoxification of DON-contaminated grains. Relaxation of the stereoselectivity, which would regenerate DON, occurred in the other studied species but not *D*. *mutans* (Fig. 6-*bottom*). Earlier investigations have reported reduced immunosuppression by *3-keto-*DON compared to DON using mitogen-induced and mitogen-free proliferations of mouse spleen lymphocytes^10^. The second step of the transformation of DON by *Devosia mutans*, namely the reduction of *3-keto-*DON to *3-epi*-DON, further reduced the toxicity 117-fold in MTT bioassays and 260-fold in BrdU bioassays^12^. These results call for the incorporation of the reduction of *3-keto-*DON into any mitigation approach of DON based on the enzymatic activities of *D*. *mutans*.

The primary mode of action of DON is the inhibition of ribosomal function^26^. Secondary toxic effects of trichothecenes have been studied for decades^2, 27^ but the molecular mechanism of protein synthesis inhibition has only recently been unraveled^16^, explaining the essential role of the epoxide group and hydroxyl group on C3 in binding to the inside the peptidyl transfer center of the ribosome. While the spiro-attachment of the epoxide group prevents conformation flexibility, the hydroxyl group on C3 was predicted by molecular modeling to exhibit significant rotation^23^ and multiple energetically equivalent conformations^24^. The loss of a hydrogen bond between the C3-hydroxyl and uracil residue 2869 of the ribosome might account for the loss of toxicity of *3-epi-*DON in comparison to DON^28^. Furthermore, the epoxide group of DON, which is not involved in intramolecular hydrogen bonds^24^, stabilizes trichothecenes in the peptidyl transfer pocket of the ribosome by hydrogen bonding with uracil 2873. The formation of an intramolecular hydrogen bond between C3 and the epoxide in *3-epi-*DON would interfere with this interaction. These structural features can explain why epimerization of DON on C3, which appears to be a minor change, causes a drastic loss of toxicity.

## Methods

### Chemicals, media formulations, and bacterial cultures

*Devosia mutans* 17-2-E-8 was isolated as reported previously^19^. Corn meal broth (CMB) was prepared as described earlier^6^ and used as the reference/propagation medium of *D*. *mutans* 17-2-E-8.

A total of 9 different carbon and nitrogen sources were further evaluated to support DON transformation where *3-keto-*DON and *3-epi-*DON metabolites were tracked. These preparations included: glucose (GLU), sucrose (SUC), corn starch (STA) as carbon sources and corn steep liquor (CSL), peptone (PEP), yeast extract (YEA), urea (URE) as organic nitrogen sources and ammonium sulfate (SUL) and ammonium nitrate (NIT) as inorganic nitrogen sources. Broths containing the above carbon or nitrogen sources (at 10 g/L) were supplemented with a mixture of minerals composed of 3 g (NH_4_)_2_SO_4_, 1 g K_2_HPO_4_, 0.5 g MgSO_4_, 0.5 g K_2_SO_4_, 0.01 g FeSO_4_, 0.007 g MnSO_4_, and 5 g yeast extract (except YEA) per 1 L of final broth. Bacterial growth was monitored as CFU^6^. DON was obtained from Sigma–Aldrich (Oakville, ON, Canada) or TripleBond (Guelph, ON, Canada). HPLC-grade methanol used for extraction was obtained from Caledon Labs (Georgetown, ON, Canada).

### Chemical synthesis of *3-keto-*deoxynivalenol

In collaboration with TripleBond (Guelph, Canada, http://www.triplebond-canada.com/), *3-keto-*deoxynivalenol (Fig. 2-*top*-*D*) was synthesized from DON (PubChem #40024) via a three-step route (Fig. 2-*top*). The major steps of this chemical synthesis were: (i) DON treatment with 10% pyridinium p-toluenesulfonate (PPTS), and 20 equiv. 2,2-dimethoxypropane in dichloromethane for 10 h at room temperature. The reaction mixture was concentrated under a reduced pressure and the dried residue was purified by flash column chromatography on silica gel to elute the isopropylidene-protected DON derivative. (ii) The oxidization step was carried out using Ley-Griffith reagent [5% tetrapropylammonium perruthenate (TPAP), 1.5 equiv. N-methylmorpholine-N-oxide (NMMO) in dicholoromethane] for 1 h at room temperature. The resulting mixture was filtered through a silica pad, concentrated, and purified through chromatography to obtain protected *3-keto-*DON. (iii) The protected *3-keto-*DON was treated with 5% trifluoroacetic acid (TFA) in dicholoromethane for 1 h at room temperature followed by chromatography purification. Proton (^1^H) NMR spectra of *3-keto-*DON (Fig. 2-*bottom*) were recorded in the Department of Chemistry, University of Guelph, ON, Canada.

### Transformation of DON by *Devosia mutans* 17-2-E-8

Transformation of DON by *D*. *mutans* 17-2-E-8 was monitored as described earlier^6^. In essence, the reduction of DON concentrations (100 μg/mL) and the formation of *3-keto-*DON and *3-epi-*DON were tracked in tubes containing 10 mL of broth inoculated with a loop of bacterial-cell suspension (1 μL). The culture was incubated aerobically at 28 °C with shaking at 150 rpm. To track DON epimerization, 100–150 µL sub-aliquots were recovered at different time points, mixed with equal volumes of HPLC-grade methanol, allowed to stand for 2 h at room temperature to maximize DON (and metabolites) extractability and centrifuged at 18,000 g for 5 min. Supernatants were submitted to LC-MS/MS analysis as described below.

In a different experiment, a seed culture (1 × 10^6^ CFU/mL based on the optical density at OD_600_) was used as inoculum for testing the effect of different carbon and nitrogen sources on transforming DON and forming *3-keto-*DON/*3-epi-*DON by isolate 17-2-E-8.

Testing mixtures (n = 4) were prepared by combining: 100 μL of the above seed culture +800 μL of the respective media +100 μL DON solution containing 1,000 μg DON/mL. Cultures were incubated aerobically for 72 h at 28 °C and shaking at 150 rpm before LC-MS/MS analysis.

### Transformations of DON and *3-keto-*DON by different *Devosia* species

Type strains representing different *Devosia* species were obtained from DSMZ (Braunschweig, Germany) and ATCC (Manassas, VA, USA) culture collections. The ability of these isolates to catabolize DON and *3-keto-*DON was investigated. *D*. *chinhatensis* (DSM24953), *D*. *soli* (DSM17780), *D*. *limi* (DSM17137), *D*. *epidermidihirudinis* (DSM25750), *D*. *subaequaris* (DSM 23447) and *D*. *riboflavina* (IFO13584) were reactivated according to the supplier’s recommended growth conditions/media (trypticase soy broth, R2A, or marine broth). The ability of such broths to support DON epimerization was confirmed using *D*. *mutans* cultures (data not shown). One mL of the actively growing culture (OD_600_ > 1) was spiked with 100 µg/mL (as final concentration) of either DON or *3-keto-*DON in triplicates and incubated at 28 °C with shaking at 150 rpm. After 24–48 h incubation, the cultures were quenched with an equal volume of HPLC-grade methanol, and shaken (120 rpm) for two hours. Bacterial pellets were removed by centrifugation and supernatants (without any further dilution) were analyzed for DON, *3-keto-*DON, and *3-epi-*DON by LC-MS/MS as described below.

### Preparation of cell-free lysate of *Devosia mutans* 17-2-E-8

One liter of LB broth was inoculated with *D*. *mutans* 17-2-E-8 and incubated at 28 °C for 5–6 days with shaking at 120–150 rpm. Bacterial pellets were collected by centrifugation at 8,400 g for 30 min and stored at −20 °C. Pellets were re-suspended in 10 mL of Tris-HCl (50 mM, pH 8.5) and a protease cocktail (Halt Protease Inhibitor, Cat. #1861278, Thermo-Scientific Canada) was added before disturbing cells through sonication. Lysates were cleared by centrifugation at 8,400 g for 1 h at 4 °C and the supernatants were filtered twice using 0.2 μm disposable filters (Whatman, 25 mm GD/X sterile, Cat. #6896-2502) and stored at −20 °C until use.

### Ammonium sulfate fractionation of protein extracts

We heated the cell-free lysates prepared as described above to 60 °C for 25 min followed by centrifugation for 30 min at 11,600 g. Ammonium sulfate solution (4.1 M) was added gradually to the supernatant to 35% saturation and the lysates were incubated on ice for two hours with shaking at 150 rpm. The precipitates were removed by centrifugation at 11,600 g for 45 min and the ammonium sulfate concentration within the remaining supernatants was further adjusted to 55% saturation. After two hours incubation on ice with continuous shaking at 150 rpm, precipitated proteins were collected by centrifugation (11,600 g, 45 min, 4 °C), re-suspended in 2–3 mL Tris-HCl buffer (50 mM, pH 8.5) and dialyzed using Slide-A-Lyzer MINI device (Thermo-Scientific, 10 K MWCO, Cat. #88404) against the same buffer (45 mL) overnight.

The dialyzed protein solution was passed through 0.2 μm disposable filters (Whatman, 25 mm GD/X sterile, Cat. #6896-2502) and the final aliquots were stored frozen at −20 °C until use.

### Cell-free enzymatic transformations of DON

Cell-free protein extracts/fractions prepared as described above were incubated with 10% glycerol, 100 μg/mL DON or *3-keto-*DON, and a source of NADP(H) at 100 μM in Tris-HCl buffer (50 mM, pH 8.5) at 37 °C. After two hours, the reactions (n = 5) were terminated by adding an equal amount of HPLC-grade methanol, the samples were cleared by centrifugation, and the supernatants were analyzed by LC-MS/MS. Ammonium sulfate fractions were tested in the same way but with overnight incubations.

### LC-MS/MS determination of DON, *3-keto-*DON and *3-epi-*DON

Liquid chromatography-tandem mass spectrometry (LC-MS/MS) analysis was carried out by using a SHIMADZU HPLC system (Kyoto, Japan) connected with a triple quadrupole IONICS 3Q Molecular Analyzer (IONICS, Bolton, Canada). The HPLC system consisted of two dual-plunger parallel-flow pumps, a membrane degasser, a column-oven, an auto-sampler, a UV/VIS photodiode array detector and a system controller. An Agilent ZORBAX SB-C18 column (2.1 × 100 mm, 3.5 µm) was used for separation. Elution gradient of solvent A (99.9% H_2_O with 0.1% formic acid) and solvent B (99.9% MeOH with 0.1% formic acid) was used as follows: 0–1 min, isocratic 10% B; 1–10 min, gradient 10% to 80% B; 10–12 min, isocratic 80% B; 12–13 min gradient 80% to 10% B; 13–18 min, isocratic 10% B. The column-oven was set to 25 °C, the flow rate was 0.4 mL/min and the injection volume was 10 µL. Electrospray in a positive mode was used for ionization. Before samples analysis, instrument parameters were optimized for DON, *3-keto-*DON and *3-epi*-DON standards at low concentrations needed for the analysis of diluted samples (samples were diluted first to the 2–10 µg/mL range before analysis). The optimized ESI conditions were as the following: the flow rates of drying gas, nebulizer gas and heating gas were 100, 390 and 350 (arbitrary units); the hot source induced de-solvation (HSID) was 280 °C; the ESI probe temperature was 300 °C; and the ion spray voltage was 5,200 V. Quantification was accomplished at multiple reaction monitoring (MRM) mode by monitoring transitions m/z 296.5 > 249.1 for DON and *3-epi*-DON and m/z 294.9 > 247.1 for *3-keto-*DON. For each metabolite the fragment ion with the highest intensity was used for quantification. The dwelling time for MRM data collection was 200 ms. Peak areas were used to establish calibration curves using least-squares regression; R^2^ was larger than 0.99 for all curves. The limits of detection of DON, *3-keto-*DON and *3-epi-*DON using this analytical procedure were around 0.001 μg/mL.

In some experiments; simultaneous determination of DON, *3-keto-*DON, and *3-epi*-DON (Fig. 3-*top*) was conducted at the Mass Spectrometry Facility within the Advanced Analysis Centre at the University of Guelph. Samples were analyzed using a Dionex UHPLC UltiMate 3000 liquid chromatograph interfaced to an amaZon SL ion trap mass spectrometer (Bruker Daltonics, Billerica, MA). Phenomenex Luna [C18(2), 5 µm, 100 Å, 150 × 2 mm] LC column was used for chromatographic separation. The initial mobile phase conditions were 95% water (0.1% formic acid) and 5% acetonitrile (0.1% formic acid) maintained for 1 min followed by a gradient to 30% acetonitrile (0.1% formic acid) in 3.5 min and the a single step gradient to 100% acetonitrile (0.1% formic acid) in 2.5 min. The flow rate was maintained at 0.4 mL/min. The mass spectrometer electrospray capillary voltage was maintained at 4.5 kV and the drying gas temperature of 280 °C with a flow rate of 10 L/min. Nebulizer pressure was 276 kPa. Nitrogen was used as both nebulizing and drying gas; helium was used as collision gas at 414 kPa. Optimal fragmentation conditions for each compound were determined using standard solutions. The mass spectrometer was set on enhanced resolution positive-ion mode. The instrument was externally calibrated with the ESI TuneMix (Agilent). MRM with the following transitions were used: 297 > 279 for *3-epi-*DON (isolation width 4), 297 > 249 for DON (isolation width 4), and 295 > 247 for *3-keto-*DON (isolation width 4). Calibration curves were created with serial dilutions of toxins standards.

### Molecular modeling of chemical structures

Hyperchem 8.0 (Hypercube Inc., Gainesville, FL, USA) was used for quantum chemical modeling of DON, *3-keto-*DON and *3-epi-*DON. The semi-empirical method of AM1 was selected for calculations and minimum-energy determinations in vacuum. The Polka-Ribiere algorithm was used for refinement with a termination threshold at RMS gradient = 0.001 kcal mol^−1^ convergence for each iteration. Gaussview 03 or PyMol^29^ were used to view simulated structures and to calculate the lengths of hydrogen bonds.

### Statistical analysis

Experiments were conducted in triplicates. Statistical inferences were established using Sigmaplot 12.5 (Systat Software Inc). Multiple group comparisons of normally distributed data were conducted by One Way Analysis of Variance (One Way ANOVA), followed by post hoc pairwise comparisons using Fisher’s protected least significant difference.
